# Neurostimulation, doping, and the spirit of sport

**DOI:** 10.1007/s12152-020-09435-7

**Published:** 2020-05-16

**Authors:** Jonathan Pugh, Christopher Pugh

**Affiliations:** 1 The Oxford Uehiro Centre for Practical Ethics, University of Oxford, Suite 8, Littlegate House, St Ebbes Street, Oxford, OX1 1PT UK; 2 Cardiff School of Sport and Health Sciences, Cardiff Metropolitan University, Cardiff, UK; 3 Cardiff Centre for Exercise and Health, Cardiff Metropolitan University, Cardiff, UK

**Keywords:** Neuro-doping, Transcranial direct current stimulation, Neurostimulation, Sport, Doping, World Anti Doping Authority, Spirit of sport, Effort, Achievement

## Abstract

There is increasing interest in using neuro-stimulation devices to achieve an ergogenic effect in elite athletes. Although the World Anti-Doping Authority (WADA) does not currently prohibit neuro-stimulation techniques, a number of researchers have called on WADA to consider its position on this issue. Focusing on trans-cranial direct current stimulation (tDCS) as a case study of an imminent so-called ‘neuro-doping’ intervention, we argue that the emerging evidence suggests that tDCS may meet WADA’s own criteria (pertaining to safety, performance-enhancing effect, and incompatibility with the ‘spirit of sport’) for a method’s inclusion on its list of prohibited substances and methods. We begin by surveying WADA’s general approach to doping, and highlight important limitations to the current evidence base regarding the performance-enhancing effect of pharmacological doping substances. We then review the current evidence base for the safety and efficacy of tDCS, and argue that despite significant shortcomings, there may be sufficient evidence for WADA to consider prohibiting tDCS, in light of the comparable flaws in the evidence base for pharmacological doping substances. In the second half of the paper, we argue that the question of whether WADA ought to ban tDCS turns significantly on the question of whether it is compatible with the ‘spirit of sport’ criterion. We critique some of the previously published positions on this, and advocate our own sport-specific and application-specific approach. Despite these arguments, we finally conclude by suggesting that tDCS ought to be monitored rather than prohibited due to compelling non-ideal considerations.

Doping is a well-documented problem in elite sport, with a considerable number of athletes employing a range of prohibited substances and methods to gain an edge in competition. However, in recent years, a new form of performance-enhancement has begun to emerge, so-called “neuro-doping”, in which athletes attempt to enhance performance by electronically stimulating the brain. As research in the area progresses, there is some emerging evidence to suggest that neuro-stimulation may have the potential to improve sporting performance by virtue of its acute effects on motor skills and cognition, and its longer tem effects on skill acquisition ([1–3). Indeed, athletes are already openly using transcranial direct current stimulation (tDCS) in training [4, 5], and researchers are also reporting ergogenic effects of trans-cranial magnetic stimulation (TMS) [6]. More speculatively, as research into non-invasive forms of Deep Brain Stimulation continues [7], the longer term future may bring far more precise forms of neuro-stimulation to bear on the enhancement of athletic performance.

Neuro-doping techniques add new dimensions to old questions about the ethics of doping in sport. Moreover, unlike many conventional forms of doping, neuro-doping techniques are currently not prohibited by the World Anti-Doping Authority (WADA). In this context, a number of researchers in the field have called on WADA to consider whether neuro-doping techniques ought to be added to its list of prohibited substances and techniques. Yet, there are a number of diverging opinions on the correct approach for WADA to take in this regard, with some researchers suggesting that current neuro-doping techniques should be prohibited by WADA [8], whilst others suggest more lenient approaches to potential regulation, including permitting its use in all sport [9], or permitting its use in training, but not in competition [10, 11].

At the outset, it is important to be clear that when considering whether any particular substance or method ought to be prohibited by WADA, the question can be asked in two senses. First, we might be interested in the question in an *ideal* sense; is the use of the substance or method compatible with the values that we want to uphold in sport? Depending on our answer, we might also be interested in the prohibition question in a *non-ideal* sense: that is, we might ask whether WADA can realistically and ethically prevent the use of a problematic doping technique in the real world. For the majority of this paper, we shall be interested in the question of whether neuro-doping should be prohibited in the ideal sense. We prioritise this question on the basis that if there is no rationale for prohibiting neuro-doping in the ideal sense, then we do not need to be concerned with the practicalities of prohibiting it. However, we shall turn to the non-idealized question in our concluding remarks.

With respect to the ideal question, we shall argue that the existing evidence-base provides a rationale that may be sufficient for WADA to prohibit some uses of neuro-doping, both in training and competition. We nuance this general position by suggesting that the variable effects that neuro-doping techniques may have call for both an application-specific and sport-specific approach to thinking about its permissibility in elite sport. To place the issue of neuro-doping into its proper context, we shall begin by surveying the approach that WADA takes to other forms of doping, and highlight important limitations to the current evidence-base for the performance-enhancing effect of currently prohibited substances. In section 2, we shall focus our discussion on tDCS as a case study of a neuro-doping intervention, and provide an overview of the current evidence base regarding its safety and ergogenic effects. We suggest that although there are significant shortcomings in the current evidence regarding the latter, the evidence base may yet be sufficient for WADA to consider prohibiting tDCS, in light of the comparable flaws in the evidence-base for other prohibited substances. As such, in the final section, we turn to the question of whether tDCS is compatible with the ‘spirit of sport’ criterion that plays a crucial role in WADA’s approach to doping. In doing so, we shall highlight some problems with some of the previously published positions on neuro-doping, and advocate our own sport-specific approach.

## WADA, doping methods and the epistemology of doping

### The WADA code & prohibited substance list

Doping carries harsh sanctions, and there are significant socio-cultural costs of being viewed as a ‘dirty’ athlete. Athletes who violate anti-doping rules are typically disqualified from the event during which the rule violation occurred, and they are often rendered ineligible from future competitions for a significant amount of time [12]. Furthermore, WADA operates a strict liability approach that allows for retrospective punishment, so athletes may be found guilty of a rule violation during and after competition, despite the absence of fault, negligence, or lack of knowledge.

Although ‘the presence or administration of a prohibited substance or method’ is not the only anti-doping rule violation [13], it is the most relevant for our purposes here. Of course, what constitutes this kind of violation depends upon what substances and methods are prohibited. These are documented in a list that is annually updated by WADA [14]. A separate document, the WADA code, includes criteria that are used to determine whether a substance or technique should be placed on the prohibited list [12]. The express purpose of the Code [12] is to bring “consistency to anti-doping rules, regulations and policies worldwide”.

Section 4.3 of the current Code states that a substance or method shall be considered for inclusion on the Prohibited List if WADA, in its sole discretion, determines that the substance or method meets any *two* of the following *three* criteria in box 1:

**Table:** Box 1

4.3.1.1 Medical or other scientific evidence, pharmacological effect or experience that the substance or method, alone or in combination with other substances or methods, *has the potential to enhance or enhances sport performance.*	
4.3.1.2 Medical or other scientific evidence, pharmacological effect or experience that the Use of the substance or method represents *an actual or potential health risk to the Athlete;*	
4.3.1.3 WADA’s determination that the Use of the substance or method *violates the ‘spirit of sport’* described in the introduction to the Code. [12]	

In the interests of brevity, we shall refer to these as the ‘enhancement’ criterion, the ‘safety’ criterion, and the ‘spirit of sport’ criterion respectively.

By virtue of the so-called ‘2/3 rule’, none of the above is a *necessary* condition of inclusion on the prohibited list. Strikingly, this means that it is possible for something to be placed on the list even if it does not satisfy the enhancement criterion. For instance, cannabinoids are included on the prohibited list [14], despite evidence suggesting that they actually diminish performance, raising considerable debate [15, 16]. This has led some to call for WADA to modify the 2/3 rule, so that the enhancement criterion becomes a necessary condition of inclusion on the prohibited list [13, 15]. Of course, if one accepts this line of argument, then this would render evidence regarding the performance-enhancing effect of any given intervention of the utmost importance in ascertaining whether or not it should be prohibited by WADA.

The enhancement and safety criteria outlined above both relate to ostensibly empirical matters. On the other hand, the spirit of sport criterion is an ethical criterion pertaining to the value and meaning of sport [17]. How should this be assessed? We shall later explain why this criterion is particularly contentious, but for now, we can make do with outlining WADA’s own understanding [12], outlined in box 2^1^:

Box 2

**Table:** 

The spirit of sport is the celebration of the human spirit, body and mind, and is reflected in values we find in and through sport, including:	
• Dedication and commitment	
• Respect for rules and laws	
• Respect for self and other participants	
• Courage	
• Community and solidarity	
• Ethics, fair play and honesty	
• Health	
• Excellence in performance	
• Character and education	
• Fun and joy	
• Teamwork [12]	

There are a few noteworthy points here. The first is that this is most charitably interpreted as a characterisation of the spirit of sport (rather than a definition), one that aims to offer only “an incomplete, unsystematic and unstructured account of key values in ethical sport”, rather than a set of necessary and sufficient conditions [15]. As McNamee [15] points out, on such a reading, some of the criticisms that have been raised against the criterion lose some of their force.^2^ That said, even on this interpretation, many of the values that constitute the characterisation are strikingly vague. McNamee has argued that this is not problematic, since we often have to accept vagueness in commonplace concepts, and that the vagueness of the spirit of sport criterion is appropriate given its role [15]. Yet, it is difficult to see how the vagueness of the criterion is not problematic *by WADA’s own lights*, for the simple reason that the express purpose of the Code is to bring consistency to anti-doping rules. Vagueness in concepts is surely the enemy of the consistent application of moral principles.

As such, it will help to elucidate the spirit of sport criterion by briefly outlining some concrete examples of the substances and techniques that WADA does (and does not) include on its Prohibited List. Doing so will also highlight the problems we face in attempting to overcome the vagueness problem, and to achieving a consistent interpretation of the criterion.

### What WADA Does (and Does Not) prohibit

There are three broad categories of substances and methods prohibited by WADA:

1. Those that are prohibited both in competition and in training;
2. Those that are prohibited only in competition;
3. Those that are prohibited only in certain sports.

Substances and techniques in the first category are placed there on the basis of their potential to enhance both acute performance and future performance. One commonly invoked example is erythropoietin (EPO), which was implicated in the Lance Armstrong doping scandal. EPO is a natural hormone that stimulates red blood cell production and decreases plasma volume. It is thought to enhance athletic performance primarily by increasing the recipient’s oxygen transport capacity, thereby delaying fatigue [22]. Other substances in this first category include, inter alia, anabolic agents, Beta 2 Agonists, and hormone and metabolic modulators.^3^ Methods in this category include, inter alia, manipulation of blood and blood components, and gene and cell doping [14].

In the second category, a smaller class of substances are banned only in completion, which (by WADA’s terms)^4^ means they are only prohibited in the twelve hours leading up to competition [12]. Substances in this category include *most* stimulants,^5^ narcotics, glucocorticoids, and cannabinoids [14]. These substances are prohibited during competition on the basis of their acute effects on performance, but they are nonetheless permitted in training.

Finally, beta-blockers are given special treatment and are included alone in the third category, as they are only banned in specific precision sports. This is largely based on evidence suggesting that they can significantly reduce tremors: One study showed that elite shooters taking beta-blockers experienced an average of 13.4% of possible improvement over placebo [23]. In the majority of precision sports, beta blockers are only prohibited in-competition (for instance in, inter alia, darts, golf, and skiing); however, they are banned both in and out of completion in the case of shooting and archery [14].

Although this exhausts the categories of substances and methods that are currently prohibited by WADA, the organisation also runs a programme to monitor the use of substances that “are not on the prohibited list, but which WADA wishes to monitor in order to detect patterns of misuse in sport” [12]. One notable inclusion on this list is caffeine. Although there is strong evidence to suggest that caffeine does have an acute performance-enhancing effect [24], it was taken off the prohibited list in 2003, partly due to difficulties in distinguishing performance-enhancing doses from normal daily consumption of coffee and soft drinks [25, 26]. Caffeine, it appears, is thus an example of a performance-enhancing agent that is not prohibited, partly on the basis of non-ideal considerations regarding the enforceability of such a ban.

There are also a number of other performance-enhancing methods and training techniques that are not prohibited or even monitored by WADA, but which serve as useful comparison cases for any theory-driven approach to moral questions about doping and the spirit of sport. For instance, the use of hypoxic air tents is permitted (albeit somewhat controversially), despite the claim that they arguably have a broadly comparable physiological effect to the prohibited substance EPO [21]. Finally, we may also note that WADA permits various kinds of expensive equipment (such as hypoxic air tents) that increase sporting performance in a manner that perpetuates unfairness in sport, not to mention the athlete’s own natural genetic constitution, which can clearly put them at an advantage over other competitors [21].

These examples of so called ‘grey zones’ [17] in the doping debate raise serious questions for how we should understand the spirit of sport criterion. Although the express aim of the WADA code is to bring consistency to anti-doping rules, it is difficult to identify a principled basis upon which we might consistently apply the spirit of sport criterion in a manner that accommodates all of these examples. We shall return to this point when we consider how WADA might accommodate neuro-doping methods. To conclude this part of the discussion though, we want to highlight an epistemological issue surrounding the general anti-doping project.

### The Epistemology of Doping

We mentioned above that some in the anti-doping literature endorse a move away from the 2/3 rule, towards one where satisfying the enhancement criterion becomes a necessary condition of inclusion on the prohibited list*.* In this context, the current evidence base for the performance-enhancing effect of substances on WADA’s prohibited list is surprisingly poor. In a recent review counting only findings from (i) double-blind, randomized controlled trials that were (ii) performed in trained subjects, and (iii) measured relevant sporting performance outcomes as evidence for performance-enhancing effects, Heuberger and Cohen [27] concluded that only five out of the 23 substance classes prohibited by WADA show evidence of having the ability to enhance actual sports performance. For instance, although EPO is often cited as a paradigmatic example of a performance-enhancing drug, these authors argue that there is a lack of high quality evidence for its performance-enhancing effects in elite athletes [27, 28].

The authors suggest that the problem with the existing evidence base is that many published studies are attended by significant methodological short-comings, when they are considered as evidence for including something on the prohibited list [27]. First, many studies use surrogate markers (such as VO_2max_) instead of direct performance measures (such as a time trial) as their outcome measure, even though the former may have low predictive value for actual athletic performance in elite athletes [27]. Second, notwithstanding concerns about the use of such surrogate markers per se, some studies use *tests* of these indirect markers that do not resemble normal exercise; for example the use of maximal exercise test to generate a VO_2max_ marker has been criticized on this score. [29]. Third, many studies do not adequately reflect the target population (namely elite and professional athletes), as they investigate the effects of substances in subjects with far lower levels of training – however, results regarding exercise interventions in one group do not necessarily translate to the other [27].

Of course, absence of evidence does not amount to evidence of absence; it may be that all of the substances on the prohibited list enhance performance, even if that has not yet been adequately empirically established. There are also a number of ethical obstacles to carrying out high quality trials of potentially harmful performance-enhancing drugs [30]. Perhaps more importantly though, the fact that WADA lacks evidence to establish that a substance or method has a performance-enhancing effect need not preclude them from prohibiting it. In some cases, this might be because there are health risks associated with the intervention in conjunction with concerns about the spirit of sport (as seems to be the case with cannabinoids). More strikingly though, given the wording of the enhancement criterion, and in particular its appeal to the mere ‘potential to enhance’, WADA is under no obligation to rely upon strong scientific evidence in supporting its decision if “sufficient determination can be found within WADA’s List Committee and Executive Committee” [13].

The safety and efficacy of a substance is an empirical matter; but the standard of evidence that WADA requires, and the thresholds it imposes in order to justify prohibiting substances, are ethical judgments. Indeed, there may be ethical reasons to require a low threshold of evidence in this context. We might invoke a precautionary principle to justify banning a substance that we have reason to suppose has the potential to pose a significant risk of harm, even if we have only limited evidence of such an effect. Similarly, if a number of athletic teams are using a substance because they believe it is effective, that itself may provide a ‘wisdom of the crowd’ evidentiary basis for precautiously assuming that the substance does have a performance-enhancing effect, even if we lack robust evidence to confirm the effect. Nonetheless, WADA has been criticized for its lack of transparency about the way it makes these sort of determinations [15, 17]. It is therefore difficult to assess how it approaches these judgments, and the broader moral and epistemological questions about how they define and empirically establish performance enhancement in sport [31].

In the interests of brevity, we must set these important questions aside, and we shall remain silent on the justifiability of the specific evidential thresholds that WADA employs. However, we outline the above considerations as they are an important lens through which we should consider the question of how we should regulate neuro-doping techniques. The key point for our purposes here is that the absence of scientific evidence for the performance-enhancing efficacy (and/or harmfulness) of an intervention is not sufficient to mean that the intervention will not fail WADA’s safety and enhancement criteria. With this in mind, we now turn our focus to tDCS.

## tDCS as a Performance-Enhancing Intervention

Researchers are currently investigating a number of different neuro-doping techniques. However, to limit the scope of our discussion, in the remainder of this paper, we shall focus on tDCS as a case study of a neuro-doping technique. We choose this specific intervention on the basis that (i) some competitors are already employing it as a training aid and (ii) there are already a considerable number of studies investigating its safety and ergogenic effect. Although we limit our discussion to this specific intervention, the way in which WADA chooses to regulate tDCS is likely to have considerable implications for how other, perhaps more powerful, neuro-doping techniques are regulated in the future.

tDCS is a non-invasive form of neuro-stimulation delivered via two electrodes placed on the scalp (incorporated into a small, portable and easily removable head-mounted device). When a weak (1-2 mA) constant current is applied for short periods of time, it passes painlessly through the brain and alters spontaneous neural activity [32]. The nature of the effects are dependent on the placement of the electrodes, as well as the magnitude and polarity of stimulation: anodal stimulation stimulates spontaneous neuronal activity, whilst cathodal stimulation inhibits it [10, 32, 33]. tDCS has already been investigated as an experimental treatment modality for neurological and psychological disorders including, inter alia, Parkinson’s Disease [34], neuropathic pain [35], and depression [36]. However, there have been mixed results in these applications, and there is still debate about the extent of tDCS’ therapeutic efficacy, possible mechanisms of any apparent therapeutic effects, ideal stimulation sites, and whether tDCS affects local stimulation sites or whole brain networks [37].

As we shall detail below, both cathodal and anodal tDCS are being investigated for their potential performance-enhancing effects. In any case, the effects of stimulation are greatest in the period of time immediately after stimulation, and decline over a period of between 20–60 min depending on the parameters of stimulation [10]. Notably, it is currently not possible to detect whether an individual has undergone tDCS, at least not without a high risk of false positive judgements [10]. This will of course have considerable implications for the non-ideal question of whether tDCS should be prohibited.

### Safety

Whilst further data is clearly necessary, existing data suggest that tDCS is a reasonably safe intervention. Like any medical procedure, tDCS is attended by some low risks of moderate adverse events (such as headaches and dizziness) [38], but when it is used within established safety parameters, these risks may be acceptably low, given the potential benefits of the technology [10, 38, 39]. A review of the safety of tDCS found that conventional uses of the intervention in human trials has not yet produced any serious adverse events [38].

Accordingly, as Davis, [10] and Imperatori et al., [11] have suggested, data regarding the safety profile of tDCS gives some grounds for doubting that tDCS would fall foul of WADA’s safety criteria for inclusion on the prohibited list.^6^ Nonetheless, the claim that tDCS is a reasonably safe intervention must be attended by the following caveats, particularly when we consider it in the context of athletic performance. The first is that we lack evidence about the long-term effects of chronic neuro-stimulation using tDCS. Second, neurophysiological studies in healthy individuals have established a short-term interaction between tDCS and pharmacological agents [40]. Although there is no evidence of these interactions leading to serious adverse effects [38], we should be mindful of the fact that we know little about the potential interactions between tDCS and other doping substances that athletes may be taking. Finally, dysfunctional tDCS devices unsurprisingly pose risks that do not attend clinical tDCS protocols [41]. This is a particularly important point in the present context, given the fact that individuals can (and do) make their own tDCS devices [42]. There is a possibility that the considerable pressure on elite athletes may tempt them to try and achieve a greater enhancing effect by using tDCS outside of established safety parameters.

Accordingly, the question of whether tDCS violates the safety criterion of the WADA code depends a great deal on the threshold of ‘potential to do harm’ that WADA employs to justify prohibition. Whilst it is plausible that tDCS could be deemed to pose a potential risk of harm that would be above a low precautionary threshold, it is unclear that employing such a low threshold is compatible with the fact that many sports put performers at significant degrees of risk [17]. For this reason, we shall simply assume that tDCS may plausibly pose a level of risk to athletes that would be acceptable to WADA. On this assumption, the question of whether WADA should prohibit tDCS turns on (i) its potential to enhance performance and (ii) whether it is compatible with the spirit of sport. In the remainder of this section we shall consider the former, before turning to the second point in more detail in the second half of this paper.

### Enhancing performance

Since tDCS can be used to modulate activity in different areas of the brain, it might plausibly have a range of effects that might be relevant for sporting performance. However, whilst some analyses of the intervention have suggested that the current evidence-base indicates that tDCS “seems to have a positive effect on exercise capacity” [1, 11], this position is not universally endorsed.

In a paper discussing the early promise of tDCS as a potential means of neuro-doping, Nick Davis [10] identified two domains in which tDCS might lead to performance enhancement in sport: (i) immediate acute gains in motor skill and (ii) longer term effects on skill acquisition. Since then, a number of detailed overviews and meta-analyses of the evolving evidence-base for the ergogenic effects of tDCS have been published ([1, 2, 3, 11, 43, 44]. Rather than repeat that work and provide an exhaustive record of the current evidence-base, here we shall give only a flavour of some of the most prominent themes in this evolving literature. We shall conclude our discussion with some critical comments on the current evidence-base, drawing on our earlier discussion of the evidence-base for substances that are currently banned by WADA.

Published studies to this point have primarily focused on the acute ergogenic effects of tDCS, and so we shall primarily concern ourselves with these effects in this overview. Prior to doing so, it should be noted that in light of evidence suggesting that tDCS can enhance skill acquisition and reproduction outside of the sporting context [45, 46], researchers have also started to investigate the effect of tDCS on the acquisition of skills required in sporting performance. Most notably, Zhu et al. [47] provided cathodal stimulation over the left dorsolateral prefrontal cortex whilst practicing a golf-putting task in a ‘training phase’. When the subjects returned for the ‘test phase’ of the experiment on another day, those in the active stimulation group performed significantly better on the golf-putting task than those in the sham group. The authors suggested that stimulation served to temporarily suppress verbal working memory activity, fostering implicit motor learning [47]. Crucially, though, the subjects had no previous golf experience, raising significant concerns about the implications of these results, particularly for elite athletic performance.

Turning to published studies on the acute effects of tDCS on motor skills, following Angius et al. [1], such studies can broadly be categorised into two groups: those that focus on the effects of tDCS on isolated muscle groups in isometric single joint exercises, and those that focus on whole body dynamic exercises. One influential study in the former category by Cogiamanian et al. [48] assessed maximum voluntary contraction (MVC) in 24 healthy subjects’ left elbow flexors and a fatiguing isometric contraction (35% of MVC), before and immediately after stimulation delivered over the cortical motor areas. Notably, their data suggest that tDCS may have led to relative improvement in muscular *endurance* in submaximal isometric contraction, but did not affect the MVC of the subjects. Further, although no evaluated electromyographic variables changed after tDCS, a significant increase in corticospinal excitability was observed. The authors took this finding to suggest the hypothesis that stimulation over the primary motor cortex serves to increase the output of this neural area during exercise, and thus modulate supraspinal fatigue.

However, further studies employing similar protocols have had only limited success in reproducing these findings. Both Kan et al. [49] and Muthalib et al. [50] employed a similar protocol but did not observe increased muscular endurance following tDCS. In contrast, Abdelmoula et al., [51] did observe increases in muscular time to fatigue but (in contrast to [48]) this occurred in the absence of increased corticospinal excitability. More recent studies have also begun to investigate the effects of tDCS in both dynamic and isokinetic exercise, again with mixed results [44, 52–54].

In the present context, an important limitation of these studies investigating isolated muscle groups is that there is not a straightforward relationship between performance on tasks performed in the studies (particularly in non-athletes) and the conditions of athletic performance [43]. In light of this, other studies investigating the acute effects of tDCS on exercise have moved their focus to its effects on whole body dynamic exercises. One of the most notable studies in this regard by Okano et al., [55] showed that peak power output of trained cyclists during a maximal incremental cycling task increased by ∼4% following anodal tDCS over the temporal cortex. This improved peak power output was also accompanied by reduced rate of perceived exertion (RPE) and heart rate during submaximal exercise intensities, compared to a sham condition.

Once again though, other comparable studies have not been able to wholly reproduce these findings. Some have failed to reproduce *any* significant ergogenic effect on whole-body exercise. Similarly applying anodal tDCS to the temporal cortex (but prior to 20-km cycling time trial rather than a maximal incremental cycling task), Barwood et al. [56] did not find any improvements in power output or cycling performance. Furthermore, Baldari et al. [57] did not find any improvement in physiological responses (including heart rate, pulmonary ventilation, and VO2_max_), perceived exertion, affective valence, or exercise performance in recreational runners on a maximal incremental running test following anodal tDCS to the primary motor cortex. In contrast Vitor-Costa et al., [58] found an improvement in submaximal exercise tolerance in physically active subjects on a cycling task following anodal tDCS to the primary motor cortex, but this was not accompanied by changes to evaluated physiological or perceptual parameters of the sort observed in Okano’s study, including the subject’s heart rate and RPE.

Finally, whilst studies investigating the acute ergogenic effect of tDCS have primarily focused on strength and endurance, a recently published study has investigated its effects on acute performance in a precision sport. Applying anodal stimulation over the cerebellar and Dorsolateral Prefrontal Cortex (DLPFC) regions, Kamali et al., [59] found that stimulation improved mean shooting score in experienced pistol shooters by 2.3%. Notably, this improvement was accompanied by a significantly decreased number of errors in a dynamic tremor task performed after the shooting task. The authors suggest that this supports a “relationship between the potentially decreased physiological tremor and enhancement in shooting performance” [59]. Although this is an isolated study, it is a notable comparator to Kruse et al.’s, [23] study (cited in the previous section) investigating the effect of Beta-Blockers on pistol shooting performance.

As even this brief overview makes clear, the evidence regarding the acute effects of tDCS on exercise performance is highly variable. Although there is some data to support the claim that tDCS may have a positive effect on exercise capacity, it is notable that two recent systematic reviews and meta-analyses have raised significant doubts about this [2, 3]. Indeed, Holgado et al. [2] conclude that “current evidence does not provide strong support to the conclusion that tDCS is an effective means to improve exercise performance”. A number of explanations for the variable results in the literature have been offered, including the fact that many studies have (i) used different stimulation parameters and montages, (ii) been statistically underpowered, (iii) measured performance on different exercise tasks, and (iv) in subjects with different levels of training [2, 3, 60].

Indeed, a study by Montenegro et al., [61] suggests that tDCS may evince favourable physiological effects amongst highly fit subjects but *not* in non-trained individuals. In this study, tDCS applied to the left temporal lobe at rest significantly increased heart rate variability amongst highly trained subjects, “enhancing the parasympathetic and decreasing the sympathetic modulation of heart rate” [61]. However, this favourable effect was not observed in non-trained individuals. This study provides reason for suggesting that Holgado et al.’s fourth explanation above is particularly significant in the context of considering WADA’s approach to tDCS.

Although these limitations to the current evidence-base suggest a need to downplay the hype surrounding the ergogenic effect of tDCS [2, 62, 63], these limitations are very similar to the those attending studies investigating the performance enhancing effect of pharmacological substances banned by WADA. As we wrote above, absence of evidence in this regard is not sufficient to preclude tDCS from failing WADA’s enhancement criterion. Accordingly, whilst it may be true that the beneficial ergogenic effects of tDCS are “largely controversial and probably more relevant in statistical rather than clinical terms” [60], that is not to say that the current evidence-base is not sufficient for tDCS to be of interest to WADA.

However, the variability of the current evidence base raises a different issue for the question of whether tDCS should qualify as a prohibited method on WADA’s terms. Even if we assume that tDCS does have a performance-enhancing effect, the conflicting results in the literature make it difficult to establish the *mechanism* via which any performance-enhancing effect is evinced [1]. Whilst this is not a problem from the perspective of the enhancement criterion, it may be from the perspective of the spirit of sport criterion. Even pre-theoretically, it seems plausible to suppose that the mechanism via which a substance or method evinces a performance-enhancing effect will have a considerable bearing on whether or not it is compatible with the spirit of sport.

This is not to say that there are no similarities across studies that can ground plausible hypotheses about potential mechanisms. Above, we described Cogiamanian et al.’s [48] hypothesis that anodal stimulation over the primary motor cortex modulates supraspinal fatigue. Whilst this hypothesis has received further empirical support in those studies where performance enhancement following stimulation is accompanied by a decrease in RPE [58], this relationship has not always been reproduced [51, 64]. Furthermore, studies finding reductions in exercise-induced pain in the absence of any improvement in performance amongst elite athletes raises the possibility of a ceiling effect on the potential of tDCS to improve motor performance in this group, even if it can nonetheless enhance affective and cognitive properties [60, 65, 66]. There is also data to suggest that such effects of stimulation on cognition and mood could offer a potential competitive advantage [67].

In view of the above discussion, the question of whether WADA ought to prohibit tDCS in light of it’s 2/3 rule will turn to a large extent on whether it is understood to violate the spirit of sport. We shall now consider this criterion.

## tDCS and the Spirit of Sport

The main challenge raised by the ‘spirit of sport’ criterion is whether it is possible to identify a principled basis that can explain WADA’s judgments about what is (and is not) included on the prohibited list, one that is also consistent with the values outlined in the spirit of sport criterion. The difficulties of meeting this challenge have led some theorists to suggest that we should abandon the criterion, and adopt revisionist approaches to the prohibition of performance-enhancing interventions [20, 21, 68]. Whatever the merits of this approach, we shall not discuss it here.^7^ Instead, we shall assume that WADA may justifiably prohibit some performance-enhancing interventions, and shall respond to some prominent attempts to provide a principled basis for interpreting the spirit of sport criterion.

To date, Imperatori et al. [11] have provided the most detailed analysis of the implications of the spirit of sport criterion for tDCS. Whilst acknowledging the considerable theoretical contribution of this article, we shall begin by critically engaging with their analysis and highlighting some points of departure.

Imperatori et al. [11] distil WADA’s spirit of sport criterion into three key features. They claim a performance enhancement is compatible with their distilled conception if it meets the following three criteria:

(i) It is sufficiently safe to use;
(ii) Hard work is required to achieve an increase in performance^8^;
(iii) It is available to most athletes.

They argue that tDCS, at least in training, is compatible with all of these criteria, and implicitly endorse the view that WADA should regulate tDCS in the same way it regulates stimulants: its use should be permitted in training but not competition. We agree with Imperatori et al. that tDCS may be sufficiently safe to use, so we shall focus our analysis on (ii) and (iii), starting with the latter.

### Inequality and Fairness

Criterion (iii) relates to conditions of social inequality, and the authors note that it is grounded by WADA’s own concern with the values of ‘respect for self and others’ and ‘community and solidarity’ in their vision of the spirit of sport (as outlined in box 1) [11]. The thought underlying this criterion is that performance-enhancing interventions should be accessible to athletes competing in the same sport. If a performance-enhancing method or substance is too expensive for most athletes to access, then its use runs contrary to the spirit of sport. However, these authors (and others) note that tDCS is compatible with this criterion because of its comparatively low financial cost (around $600 per device) [11, 69].

We agree with Imperatori et al. that tDCS is reasonably accessible as an ergogenic aid from a financial perspective. However, there is scope for questioning whether financial inequality is the *only* consideration relevant to considerations of fairness in this context.

In many ways, sport is a celebration of certain inequalities [70]. Imperatori et al. [11] also specifically advert to the fact that athletic performance is heavily influenced by factors that are largely outside of the athlete’s control, including financial investment and the individual’s athletic traits. They argue that a point in favour of tDCS is that it may be used to somewhat level the playing field between amateur athletes and elite athletes by ‘steepening the learning curve’ for amateurs, and reducing the effects of some of these inequalities.^9^

These authors all argue that we should try to limit the influence of certain inequalities in sport. But how do we distinguish those inequalities that are compatible with fair competition, and those whose influence we ought to diminish? Although they do not directly consider this question, the fact that Imperatori et al. [71] note that athletes lack *control* over certain key factors that impact performance suggests that they may take control to be a morally relevant factor here. If so, they may endorse the view that we should seek to diminish sources of inequality that lie outside of the individual’s control, a position recently defended in more detail by Loland [71].

We shall engage with the position in further detail below, as it is crucially linked to Imperatori et al.’s hard work condition. At this point though, we may note that even assuming this understanding of the normative significance of inequality in sport (and we shall raise some doubts about it below), a problem with invoking it in defence of tDCS is that this intervention might *introduce* forms of inequality that athletes cannot control, even though it may reduce the influence of others. As Lavazza [9] points out, data from studies investigating the effect of tDCS on cognition suggests that the effects of neuro-stimulation are highly variable between subjects, with some individuals failing to experience any enhancement effect from stimulation. If, as the current evidence seems to suggest, this variability is attributable to individual differences in neuroanatomy (broadly construed) and genetic characteristics (rather than electrode placement and stimulation parameters alone) [9], it is plausible that further investigation may show a similar inter-individual variability in the ergogenic effect of tDCS.

If this is the case, then tDCS could plausibly be said to provide an unequal advantage to those athletes who are responders – yet, since the individual athlete can exert little control over whether she is a responder, it seems that this is just the sort of inequality that we should be trying to reduce by Imperatori et al.’s own lights.

This is not intended to be a knock-down argument against the claim that tDCS is compatible with fairness in sport. For instance, one might hold that the variable effects of tDCS introduce inequalities that can be compensated in other ways [9]. Alternatively, one might claim that the inter-individual variable effects of tDCS are not normatively significant, even though the influence of this sort of inequality lies outside of the athlete’s sphere of control. Indeed, as we shall go on to discuss, recent discussions of the spirit of sport criterion may offer a theoretical basis for this argumentative strategy. The two salient points here though are that (i) performance-enhancing strategies may be inaccessible for some athletes for non-financial reasons and (ii) appeals to control may not be sufficient to explain why these forms of inaccessibility are not normatively significant from the perspective of fairness. To go deeper into this latter point, we can turn to considerations raised by Imperatori et al.’s criterion of hard work. For the sake of argument, we shall now set these concerns about fairness per se to one side, and assume that the performance enhancing effects of tDCS are accessible to all athletes.

According to these authors, hard work is defined as “Training the physical skill that is tested in competition, which contributes to the ‘development of the whole human” [11]. In order to test whether the use of a substance or method is compatible with hard work so defined, they suggest that we have to ask whether its use enables those who are sedentary to gain a significant, sustained, and cumulative performance enhancement. The thought here seems to be that if a substance or method does provide such an advantage to sedentary individuals, then it is not compatible with hard work; such evidence would suggest that individuals using the substance gain a long-term advantage *in the absence of training*. Instead, the advantage would reflect long-term changes in the individual’s physiology [11].

By way of illustration, the authors suggest that EPO would fail this test, but that tDCS would pass it. The authors point out that a study by Sieljacks et al., [72] suggests that a 10-week programme of EPO administration to sedentary individuals led to significant relative increases in VO_2max_ compared to sham control groups, even when controlling for the effects of training. Accordingly, Imperatori et al. [11] suggest that this study indicates that “. . . EPO administration can clearly increase performance without the need to train, at least in non-athletes”. Furthermore, they also note that a similar effect has been observed in trained cyclists [28].^10^ In contrast, they suggest that “training is crucial to making significant changes in performance using tDCS” [11].^11^

Whilst this is generally true, Imperatori et al.’s argument here moves too quickly. It is true that the putative long-term enhancing effect of tDCS on skill acquisition and retention would require the athlete to train the skill in question. However, as we discussed above, there is some evidence to suggest that tDCS may also have *acute* physiological effects that may be relevant to athletic performance. Crucially, studies in which these acute effects have been observed have not incorporated a training period into their design [55, 58]. Moreover, even if these effect sizes are comparatively small, seemingly trivial improvements may be sufficient to have an important influence on elite sporting outcomes [3, 60].^12^ It is thus unclear how these acute effects would be compatible with Imperatori et al.’s hard work criterion. Indeed, Imperatori et al. themselves seem to implicitly acknowledge this point (despite their remarks quoted above), since they conclude their analysis by arguing that tDCS should be permitted in training only, and not in competition [11]. They later clarify that their position is intended to be implemented in all sports [11].

We are sympathetic to the general contours of Imperatori’s conclusion here. If the positive data in favour of the ergogenic effects of tDCS is correct, then tDCS could plausibly have acute effects on sporting performance that would be comparable in performance-enhancing effect (if not in mechanism) to other banned substances and methods. Moreover, depending on the mechanism via which such effects are evinced, it might plausibly do so in a manner that would undermine the contribution of ‘hard work’ to performance. Finally, we also agree with them to the extent that the use of tDCS in training may not always be problematic. However, we also disagree with some points of their analysis and their adoption of a one-size fits all approach to regulating tDCS in sport.

To see why, we need to explain some problems with Imperatori et al.’s analysis of the role of hard work in the spirit of sport. First, in some ways, their interpretation of the criterion is too broad; it would lend support to prohibiting a number of methods that are currently permitted. Consider first their emphasis on ‘the physical’ in delineating the nature of hard work. One problem with this is that there are a number of ways in which individuals can legitimately increase performance without training a *physical* skill. Indeed, the whole enterprise of sports psychology is founded on this notion. The mere fact that interventions in sports psychology do not involve training a physical skill does not mean that they are thereby incompatible with hard work, or that they are thereby contrary to the spirit of sport. This is particularly notable in the present context, in view of the aforementioned data suggesting that tDCS may have beneficial effects on elements of mood and cognition that are evinced in the absence of training a physical skill, but which may nonetheless have an impact on athletic performance.

It is also difficult to see how the use of hypoxic air tents could satisfy the hard work criterion, despite the fact that WADA permits their use. Imperatori et al. attempt to circumvent this particular criticism by claiming that the hard work criterion does not apply to practices that are essential to survival (such as breathing) or artificial means of controlling or optimising these essential practices, because this could conflict with their first criterion of safety [11]. However, this is unconvincing; as WADA themselves concluded after investigating the matter, hypoxic air tents do not pose an unreasonable safety risk if used within established parameters and under medical supervision [74, 75].

Whilst the above considerations suggest that Imperatori’s et al.’s conception of the hard work criterion is overly broad, this does not directly jeopardise their conclusion that tDCS is compatible with the spirit of sport. Rather, assuming the authors do not want to endorse a revisionist stance to the use of sports psychology and hypoxic air tents, it suggests that further elements need to be built into their conception of ‘hard work’ in order to explain why these interventions should be permitted alongside tDCS, whilst others (EPO for example) should still be prohibited. However, the second set of concerns we shall now raise does put pressure on the conclusion that tDCS should be permitted in training, since we shall now suggest that the hard work criterion is also too narrow. Contrary to Imperatori et al.’s analysis, even in conjunction with considerations of safety and accessibility, the hard work criterion may not capture all of the interventions that might plausibly be deemed to be contrary to the spirit of sport.

To begin, we may note a problem with Imperatori et al.’s test for whether a substance or method obviates the need for hard work. Recall that their test for this is to ask whether that substance or method enables sedentary individuals to gain a significant sustained, cumulative performance enhancement. Whilst this approach might serve to isolate the contribution that an enhancing intervention is making to performance in these individuals, the test is nonetheless problematic if its conclusions are generalised to justify prohibiting elite athletes from using an intervention like tDCS. As we emphasised above, there are a number of reasons why results in studies of exercise outcome measures in sedentary individuals will not transfer to elite athletes. Indeed, as we described above, Montenegro et al.’s [61] data suggesting that tDCS improved heart rate variability in athletes but not in non-athletes, should give us caution in this regard. This is only one study in the specific context of the ergogenic effects of tDCS, but it suggests that we should be careful of using Imperatori’s test concerning results in sedentary individuals to ascertain when a performance-enhancing substance is reducing the need for hard-work in elite athletes. The test may not be capturing what is morally significant by their own lights.

But a perhaps more striking feature of Imperatori at al’s appeal to the hard work criterion is that it is unclear *why* they (or by proxy WADA) think that hard work should matter. The authors suggest that hard work has occurred if ‘training is required’ to evince performance enhancement. But this alone is not a sufficient answer to the question at hand – training can make more or less of a contribution to achieving a better performance in future competition, and it might matter in very different ways. Indeed, a substance or method could radically amplify the effect that training has on future performance. For instance, whilst steroids can have acute effects in the absence of training, data suggest that they have far greater ergogenic effects when they are used in combination with training [73]. In a sense then, training is required to get the most powerful ergogenic effects of steroids – but it is not clear that this should mean that steroid use in combination with training is compatible with the hard work criterion, but their use in the absence of training is not.

As such, even assuming that some of the ergogenic effects of tDCS require training, a crucial remaining question is what sort of influence tDCS has on the effects of such training. In addition to this empirical question, Imperatori et al.’s position raises the further question of how we should think about the moral significance of training, and its implications for the longer-term effects of tDCS. Their appeal to considerations of control in their discussion of inequality offer some clues about why they take training to matter morally, echoing Sigmund Loland’s recent analysis of the matter.

Loland [71] argues that bans on doping can be justified by appealing to ideals of natural human performance and the normative structure of sport. He argues that performance enhancement evinced by training is compatible with natural human performance, in so far as it involves the systematic utilization of adaptive processes that we humans have developed over the course of evolution, and which demarcate the boundaries of “physiological authenticity” [71]. In contrast, doping substances (such as EPO) involve bypassing (rather than merely exploiting) these natural adaptive processes; they thus cannot be said to contribute to a performance that is authentic in this particular sense.

On a simplistic analysis (which Loland himself does not endorse), one might say that training is significant just because of its compatibility with natural human performance, so construed. Such an approach could spell trouble for the use of tDCS in training, if the mechanism via which tDCS provides an ergogenic effect bypasses the kinds of adaptive processes that Loland champions. However, notwithstanding the difficulties with empirically establishing this, the challenge facing this simplistic approach is that it clearly courts the naturalistic fallacy; why should we suppose that ‘the natural’ is good in this context? Loland circumvents this problem by offering a deeper analysis, according to which the significance of safeguarding the natural in training can be grounded by considerations of fairness. He argues that sport is normatively structured to reduce as far as possible, inequalities over which the competitor has no control. Although inequalities in athletic ability are part and parcel of sport, he suggests that these can be fitted within its normative structure, because these differences are largely attributable to the athlete’s training and effort, and therefore his control [17, 71].

Loland’s analysis has clear affinities with some of Imperatori’s remarks about the significance of control to considerations of inequality and fairness. Moreover, the approach seems to lend support to the permissibility of using tDCS to enhance the effect of training on skill acquisition and training, as Imperatori suggest. However, whilst it might be plausible to suppose that an appeal to considerations of control can ground the normative significance of ‘physiological authenticity’ and training, the empirical premises of Loland’s argument here seem highly doubtful – First, it is highly doubtful that most inequalities in athletic ability are largely attributable to the athlete’s training and effort. We can exert only limited control over many of the key physical attributes (such as height) that are central to success in many sports, and it is not clear that these inequalities are adequately compensated for by those features that we can control [76, 77].

Second, there is the still deeper issue of why we should assume that the individual is responsible for the results they garner from training, or indeed the degree of effort they exert in training. These too may depend on elements that the individual herself does not wholly control, such as her native ability to exert effort and will-power [78]. Various elements that the individual does not control plausibly contribute to these features, including the individual’s genetic make-up, education, and social environment. With respect to genetics, Timmons et al. [79] have shown that RNA profiling and single-gene DNA marker association analysis can be used to yield biomarkers with high predictive power about an individual’s VO_2max_ response to endurance training. Although this evidence is not conclusive, it is sufficient to at least raise doubts about the assumption that the individual is wholly responsible for the results they garner from training.

Accordingly, we believe that there are significant problems with attempting to flesh out the normative structure of sport by appeal to considerations of the natural, control, responsibility, and fairness. However, there is an alternative virtue-based view (developed in most detail by McNamee [80]) about the normative structure of sport that is of use here. Instead of claiming that the effort exerted in training is key to its normative significance because it is natural and/or the individual *controls* the degree of effort they exert, one might instead claim that experiencing the adversity of expending effort just is constitutive of a central *virtue* that certain sports are intended to promote and celebrate. The thought here is that the display of certain virtues, and the ability to endure particular kinds of costs, is plausibly central to the excellences that largely define the nature of different sports [81].

Notably, Savulescu, who adopts a revisionist approach to doping in sports, has argued with colleagues that the praiseworthiness of an agent for some endeavour (outside of the sporting context) depends on (i) the voluntariness and strength of the agent’s committed pursuit of a valuable end (E), (ii) the value of E and (iii) the costliness of the committed pursuit of E [82]. They note that the expenditure of effort is just one kind of (fungible) cost. Contrary to this analysis though, we claim that not all kinds of costs are fungible in the specific context of sport. Consider for instance, long-distance cycling; the adversity of the exertion of effort involved in this sport (both in and out of competition) is not just one cost that could be exchanged for others (such as exposing oneself to a risky procedure that would eliminate the experience of pain) in a way that would safeguard the individual’s praiseworthiness for the achievement of winning a long-distance race. Rather, if you take away this particular kind of cost and the excellence involved in overcoming it, you are no longer talking about the same sport; to rework the phrase, in some cases, if there is no pain, its not the same game.^13^

What is the upshot of this kind of view for answering the ideal question concerning the use of tDCS in training? We can only offer a brief sketch here, but contrary to Imperatori at al.’s one size fits all approach to discussing the effects of tDCS in sport, this view calls for a nuanced approach to the different effects that tDCS might evince, the mechanisms by which it might do so, and also the particular sport in question.^14^

First, the view we have outlined above places a great deal of importance on the different excellences that are central to different sports. Suppose that tDCS were used to reduce the adversity of training through diminishing the training athlete’s rate of perceived exertion. If so, tDCS would plausibly serve to diminish the extent to which an athlete exhibits the particular kind of virtue involved in overcoming such adversity in training. However, even assuming that these beneficial training effects would carry over to performance in competition, this does not alone entail that it is contrary to the spirit of all and every sport. On the view we are appealing to here, sports are organized so that “different excellences contribute to the outcome of sporting competition to different degrees” [81]. In some sports then, such as endurance cycling, displays of prolonged and repeated physical exertion in training are plausibly a central excellence of the sport; in others, it may be more plausible to suggest that the need for such displays of exertion in training is in fact a barrier to the athlete developing skills required by other excellences that ought to have greater weight in determining outcomes in that sport. We might, for instance, want to place greater emphasis on a footballer’s ball skills and creativity than their ability to cope with tests of physical endurance in long training sessions, or matches that go into extra time. In a similar vein, the virtue involved in spending time engaging in repetitive training exercises to engrain muscle memory on a very specific motor skill may be a peripheral excellence to some sports. Recalling that we are assuming fair universal access to the benefits of tDCS for the sake of argument here, our point is that if the enhancement of skill acquisition and retention that tDCS might evince enables athletes to spend less time on such exercises, and more time developing skills that are required by other excellences in the sport, then it may allow athletes to come closer to practicing and performing in accordance with the true ideals of the sport in question.

In reality, this debate about the normative significance of training reflects a deeper conflict about the nature and value of sport: Do we want sport to celebrate the natural, do we want to create a level playing field that prioritizes the athlete’s control over the outcome of competition,^15^ or do we want to celebrate the particular kind of excellence and virtues associated with different sports? We cannot hope to definitively answer this question here - but the answer we give will have implications for whether we should understand the effect that tDCS might have on training to be compatible with the deep values that we take the spirit of sport to signify. In any case, contrary to Imperatori’s analysis, simply adverting to the fact that the performance-enhancing effects of tDCS are only achieved through training does not exhaust the moral question of whether its use in training is compatible with the sort of hard work that they take the spirit of sport to necessitate.

Two final concrete casuistic observations are apposite. First, recall that Imperatori et al. suggest that tDCS should be treated by WADA in the same way as stimulants (i.e. prohibited only in competition). However, it is by no means clear that stimulants and tDCS would have comparable effects on training. If tDCS is to be treated on the same terms as stimulants on this score, then it is crucial that we obtain further data to investigate the long-term effects of tDCS-assisted training on later performance, in comparison to the long-term effects of stimulant-assisted training. Second, emerging data regarding the effects of tDCS on precision sport performance [59] suggests that consistency may demand that WADA apply the same sort of sport-specific approach it takes to out-of competition tDCS as it does to Beta-Blockers. Recall that WADA prohibits the use of beta-blockers for a number of precision sports. If tDCS and Beta Blockers meaningfully improve performance in precision sport by reducing tremors (as the limited data at least suggests), then they ought to be included in the same sport-specific categories of the prohibited list for these uses.

## Conclusion – From the Ideal to the Non-Ideal.

Across the whole doping debate there is significant disagreement about how we should interpret the WADA criteria for inclusion on the Prohibited List. Yet, even across divergent interpretations, there are some broad areas of agreement. Only those who adopt highly revisionary approaches to anti-doping would deny that drugs with apparently significant ergogenic effects and significant potential of harm should be prohibited. Furthermore, there are certain means and methods of improving performance through standard forms of training that are not only clearly permissible, but which may partly constitute a paradigmatic expression of the spirit of sport. The main area of debate in this context pertains to the ‘grey zone’ between these two extremes, where we must consider interventions that pose acceptable levels of risk and which may have some performance-enhancing effect, but an effect that does not rely solely on the athlete’s extant physical and mental attributes.

tDCS lies firmly in this grey zone; however, we have suggested that there are grounds for supposing that certain applications of tDCS could plausibly be compatible with the spirit of sport, construed on an ‘excellence-based’ understanding. However, contrary to Imperatori et al., even assuming the safety of tDCS, we have suggested that considering whether (i) tDCS is financially accessible and (ii) whether its enhancing effect requires training is not alone sufficient to adequately answer the question of whether it is compatible with the spirit of sport. We have suggested that there is a need for a more nuanced approach that is sensitive to the different effects that tDCS might evince, the mechanisms by which it might do so, and the excellences and virtues that we want to emphasise in different sports, both in and out of competition.

Our discussion in this paper has pertained to the *ideal* question of whether tDCS should be prohibited; we believe that our discussion suggests that there is a clear need to also consider the non-ideal question of whether and how it could be practicable for WADA to prohibit certain uses of tDCS. When we turn to the non-ideal question, a number of further concrete facts about tDCS become more salient than abstract discussions of the spirit of sport. First, it is currently extremely difficult to accurately test whether an individual has undergone tDCS [10]; this problem might also be compounded by legitimate potential future therapeutic uses of neuro-stimulation, particularly to treat mild traumatic brain injuries and concussion that may result from participation in certain sports [85–88] .

Yet, difficulty of detection alone is arguably not sufficient to justify refraining from prohibiting a performance-enhancing intervention; for instance, the use of xenon and argon is also banned by WADA, despite concerns about the absence of a valid test for these substances [89]. Indeed, there can perhaps be some value in prohibiting methods and substances that one cannot detect, in order to communicate social condemnation of those practices. However, in the case of tDCS, in addition to the difficulty of detection, we also know relatively little about its safety and efficacy profile; but we *do *know that poorly designed dysfunctional versions of the technology pose a greater safety risk, and that it is possible for individuals to build their own devices.

In our view, these considerations cumulatively speak in favour of listing tDCS as a method that should *not* be prohibited by WADA, given the nature of our non-ideal circumstances. Contrary to Imperatori et al.’s analysis, the relevant comparator for the *regulation* of tDCS in the non-ideal context should not be stimulants per se; rather it should be caffeine. As discussed above, caffeine was taken off the prohibited list in light of non-ideal considerations, and we suggest that WADA should adopt a similar approach to tDCS as it currently does to caffeine. Even if we assume there are strong ideal grounds for prohibiting tDCS in competition, it is difficult to see how such a ban could be enforced, and prohibition could encourage use the use of unsafe devices or stimulation parameters. As such, we suggest that WADA should monitor the use of tDCS in the same way that caffeine is currently monitored. As part of that monitoring, WADA should work with companies developing tDCS devices to ensure that athletes are properly informed about the effects and risks of tDCS. Athletes should be encouraged to report their use of tDCS, and to participate in controlled studies, so that we might better understand the nature of tDCS’ effects in this specific cohort, and to attain the quality evidence that is missing in pharmacological doping. Such studies are central to ensuring that athletes now and in the future remain properly informed about the risk/benefit profile of tDCS, and to fully understanding whether it, and future forms of neuro-doping, will truly violate the spirit of sport.

## Footnotes

1 For a proposed revision, see (Loland and McNamee, [17]

2 For such criticisms, see [13, 18–21]. Some of these authors use these criticisms of the spirit of sport criterion as a basis to develop highly revisionist approaches to anti-doping rules. Here though, we shall only be interested in the question of whether tDCS should be banned according to the WADA criteria.

3 Diuretics and masking agents are also prohibited both in and out of competition, due to non-ideal considerations about enforcing the prohibition of other enhancing interventions.

4 The WADA code provisions allow for an International Federation or ruling body of particular events to offer alternative definitions of ‘in-competition’ [12].

5 Clonidine and Imidazole are excluded from this prohibition. So too are 7 stimulants that are included in the 2019 Monitoring Program Bupropion, caffeine, nicotine, phenylephrine, phenylpropanolamine, pipradrol, and synephrine.

6 For a dissenting view, see [8]

7 Similarly, we shall not engage further with Lavazza’s [9] compensation strategy for tDCS in sport, which is premised on the thought that inequalities raised by tDCS could be compensated for by allowing athletes to use other drugs prohibited by WADA.

8 Imperatori et al. [11] take this criterion to be implied by WADA’s appeal to ‘character and education’ and ‘dedication and commitment’ in their Spirit of Sport characterization.

9 Whilst we have doubts about the extent to which tDCS could level the playing field in this way, let us put that empirical point to one side.

10 For data suggesting that steroids can similarly have a performance-enhancing effect without the need for training see [73]

11 Sampedro and Triviño, (2017) similarly emphasize this point.

12 For this reason, we disagree with Sampedro and Pérez Triviño’s [69] suggestion that neuro-doping is likely to be unproblematic because it will only lead to small differences in results.

13 For a specific discussion of the nature and values inherent in endurance sport, see [80, 83]. Notably, there is evidence to suggest that EPO, the paradigm case of a doping substance, increases an individual’s rate of perceived exertion whilst it enhances performance; that is, it may make the enhanced performance feel more difficult [84].

14 Davis [10] also supports a sports specific approach to regulating neuro-doping.

15 Alex Erler [76] convincingly argues that these two aspects are fundamentally in conflict.
